# Supplementary material for: The Role of the Gut and Airway Microbiota in Chronic Rhinosinusitis with Nasal Polyps: A Systematic Review
Source: Int J Mol Sci. 2024 Jul 27;25(15):8223. doi: 10.3390/ijms25158223 (PMC11311313; doi:10.3390/ijms25158223)
Supplement: Supplementary file 1 [file ijms-25-08223-s001.zip › Supplementary Table S1_26062024.pdf]

1. Supplementary Material

Supplementary Table S1. Search strategy for each online database.

	Query	Results (05 Feb 2024)
Pubmed		
#1	"crswnp"[All Fields] OR "crswnps"[All Fields] OR (("chronic"[All Fields] OR "chronical"[All Fields] OR "chronically"[All Fields] OR "chronicities"[All Fields] OR "chronicity"[All Fields] OR "chronicization"[All Fields] OR "chronics"[All Fields]) AND ("rhinosinusal"[All Fields] OR "rhinosinusitis"[MeSH Terms] OR "rhinosinusitis"[All Fields]) AND ("nasal polyps"[MeSH Terms] OR ("nasal"[All Fields] AND "polyps"[All Fields]) OR "nasal polyps"[All Fields])) OR ("nasal polyps"[MeSH Terms] OR ("nasal"[All Fields] AND "polyps"[All Fields]) OR "nasal polyps"[All Fields] OR ("nose"[All Fields] AND "polyp"[All Fields]) OR "nose polyp"[All Fields]) OR ("nasal polyps"[MeSH Terms] OR ("nasal"[All Fields] AND "polyps"[All Fields]) OR "nasal polyps"[All Fields]) OR ("nasal polyps"[MeSH Terms] OR ("nasal"[All Fields] AND "polyps"[All Fields]) OR "nasal polyps"[All Fields] OR ("nasal"[All Fields] AND "polyp"[All Fields]) OR "nasal polyp"[All Fields]) OR ("nasal polyps"[MeSH Terms] OR ("nasal"[All Fields] AND "polyps"[All Fields]) OR "nasal polyps"[All Fields] OR ("polyp"[All Fields] AND "nasal"[All Fields]) OR "polyp nasal"[All Fields]) OR ("nasal polyps"[MeSH Terms] OR ("nasal"[All Fields] AND "polyps"[All Fields]) OR "nasal polyps"[All Fields] OR ("polyps"[All Fields] AND "nasal"[All Fields]) OR "polyps nasal"[All Fields]) OR ("nasal polyps"[MeSH Terms] OR ("nasal"[All Fields] AND "polyps"[All Fields]) OR "nasal polyps"[All Fields] OR ("nasal"[All Fields] AND "cavity"[All Fields] AND "polyp"[All Fields]) OR "nasal cavity polyp"[All Fields]) OR ("nasal polyps"[MeSH Terms] OR ("nasal"[All Fields] AND "polyps"[All Fields]) OR "nasal polyps"[All Fields] OR ("nasal"[All Fields] AND "polyposis"[All Fields]) OR "nasal polyposis"[All Fields]) OR (("nose"[MeSH Terms] OR "nose"[All Fields]) AND "polyposis"[All Fields]) OR (("nasal polyps"[MeSH Terms] OR ("nasal"[All Fields] AND "polyps"[All Fields]) OR "nasal polyps"[All Fields] OR ("polyp"[All Fields] AND "nasal"[All Fields]) OR "polyp nasal"[All Fields]) AND ("cavity s"[All Fields] OR "dental caries"[MeSH Terms] OR ("dental"[All Fields] AND "caries"[All Fields]) OR "dental caries"[All Fields] OR "cavities"[All Fields] OR "cavity"[All Fields])) OR (("polyp s"[All Fields] OR "polypous"[All Fields] OR "polyps"[MeSH Terms] OR "polyps"[All Fields] OR "polyp"[All Fields]) AND ("nose"[MeSH Terms] OR "nose"[All Fields])) OR ("nasal polyps"[MeSH Terms] OR ("nasal"[All Fields] AND "polyps"[All Fields]) OR "nasal polyps"[All Fields] OR ("polyposis"[All Fields] AND "nasi"[All Fields]) OR "polyposis nasi"[All Fields]) OR ("polyposis"[All Fields] AND ("nose"[MeSH Terms] OR "nose"[All Fields])) OR ("sino-nasal"[All Fields] AND ("polyp s"[All Fields] OR "polypous"[All Fields] OR "polyps"[MeSH Terms] OR "polyps"[All Fields] OR "polyp"[All Fields]))	12,536
#2	"microbiota"[MeSH Terms] OR "microbiota"[All Fields] OR "microbiotas"[All Fields] OR "microbiota s"[All Fields] OR "microbiotae"[All Fields] OR ("microbiota"[MeSH Terms] OR "microbiota"[All Fields] OR "microbiotas"[All Fields] OR "microbiota s"[All Fields] OR "microbiotae"[All Fields]) OR ("microbiota"[MeSH Terms] OR "microbiota"[All Fields] OR ("microbial"[All Fields] AND "community"[All Fields]) OR "microbial community"[All Fields]) OR ("microbiota"[MeSH Terms] OR "microbiota"[All Fields] OR ("community"[All Fields] AND "microbial"[All Fields]) OR "community microbial"[All Fields]) OR ("microbiota"[MeSH	548,248

Terms] OR "microbiota"[All Fields] OR ("microbial"[All Fields] AND "communities"[All Fields]) OR "microbial communities"[All Fields]) OR ("microbiota"[MeSH Terms] OR "microbiota"[All Fields] OR ("microbial"[All Fields] AND "community"[All Fields] AND "composition"[All Fields]) OR "microbial community composition"[All Fields]) OR ("microbiota"[MeSH Terms] OR "microbiota"[All Fields] OR ("community"[All Fields] AND "composition"[All Fields] AND "microbial"[All Fields]) OR "community composition microbial"[All Fields]) OR ("microbiota"[MeSH Terms] OR "microbiota"[All Fields] OR ("composition"[All Fields] AND "microbial"[All Fields] AND "community"[All Fields]) OR "composition microbial community"[All Fields]) OR ("microbiota"[MeSH Terms] OR "microbiota"[All Fields] OR ("microbial"[All Fields] AND "community"[All Fields] AND "compositions"[All Fields]) OR "microbial community compositions"[All Fields]) OR ("microbiota"[MeSH Terms] OR "microbiota"[All Fields] OR ("microbial"[All Fields] AND "community"[All Fields] AND "structure"[All Fields]) OR "microbial community structure"[All Fields]) OR ("microbiota"[MeSH Terms] OR "microbiota"[All Fields] OR ("community"[All Fields] AND "structure"[All Fields] AND "microbial"[All Fields]) OR "community structure microbial"[All Fields]) OR ("microbiota"[MeSH Terms] OR "microbiota"[All Fields] OR ("microbial"[All Fields] AND "community"[All Fields] AND "structures"[All Fields]) OR "microbial community structures"[All Fields]) OR ("microbiome s"[All Fields] OR "microbiomic"[All Fields] OR "microbiomics"[All Fields] OR "microbiota"[MeSH Terms] OR "microbiota"[All Fields] OR "microbiome"[All Fields] OR "microbiomes"[All Fields]) OR ("microbiome s"[All Fields] OR "microbiomic"[All Fields] OR "microbiomics"[All Fields] OR "microbiota"[MeSH Terms] OR "microbiota"[All Fields] OR "microbiome"[All Fields] OR "microbiomes"[All Fields]) OR ("microbiota"[MeSH Terms] OR "microbiota"[All Fields] OR ("human"[All Fields] AND "microbiome"[All Fields]) OR "human microbiome"[All Fields]) OR ("microbiota"[MeSH Terms] OR "microbiota"[All Fields] OR ("human"[All Fields] AND "microbiomes"[All Fields]) OR "human microbiomes"[All Fields]) OR ("microbiota"[MeSH Terms] OR "microbiota"[All Fields] OR ("microbiome"[All Fields] AND "human"[All Fields]) OR "microbiome human"[All Fields]) OR ("microflora"[All Fields] OR "microflorae"[All Fields] OR "microfloras"[All Fields]) OR (("microbial"[All Fields] OR "microbially"[All Fields] OR "microbials"[All Fields]) AND ("flora"[All Fields] OR "florae"[All Fields] OR "floras"[All Fields])) OR (("bacterial"[All Fields] OR "bacterially"[All Fields] OR "bacterials"[All Fields]) AND ("flora"[All Fields] OR "florae"[All Fields] OR "floras"[All Fields])) OR (("bacterial"[All Fields] OR "bacterially"[All Fields] OR "bacterials"[All Fields]) AND ("microbiota"[MeSH Terms] OR "microbiota"[All Fields] OR "microbiotas"[All Fields] OR "microbiota s"[All Fields] OR "microbiotae"[All Fields])) OR (("bacteriologic"[All Fields] OR "bacteriological"[All Fields] OR "bacteriologically"[All Fields]) AND ("flora"[All Fields] OR "florae"[All Fields] OR "floras"[All Fields])) OR (("bacteria"[MeSH Terms] OR "bacteria"[All Fields] OR "bacterium"[All Fields] OR "bacterium s"[All Fields]) AND ("flora"[All Fields] OR "florae"[All Fields] OR "floras"[All Fields])) OR (("infant, newborn"[MeSH Terms] OR ("infant"[All Fields] AND "newborn"[All Fields]) OR "newborn infant"[All Fields] OR "neonatal"[All Fields] OR "neonate"[All Fields] OR "neonates"[All Fields] OR "neonatality"[All Fields] OR "neonatals"[All Fields] OR "neonate s"[All Fields]) AND ("bacterial"[All Fields] OR "bacterially"[All Fields] OR "bacterials"[All Fields]) AND ("flora"[All Fields] OR "florae"[All Fields] OR "floras"[All Fields])) OR (("bowel s"[All Fields] OR "bowell"[All Fields] OR "intestines"[MeSH Terms] OR "intestines"[All Fields] OR "bowel"[All Fields] OR "bowels"[All Fields]) AND ("microbiota"[MeSH Terms] OR "microbiota"[All Fields] OR "microbiotas"[All Fields] OR "microbiota s"[All Fields] OR "microbiotae"[All Fields]))

OR (("gastrointestinal tract"[MeSH Terms] OR ("gastrointestinal"[All Fields] AND "tract"[All Fields]) OR "gastrointestinal tract"[All Fields] OR ("alimentary"[All Fields] AND "canal"[All Fields]) OR "alimentary canal"[All Fields]) AND ("flora"[All Fields] OR "florae"[All Fields] OR "floras"[All Fields])) OR (("gastrointestinal tract"[MeSH Terms] OR ("gastrointestinal"[All Fields] AND "tract"[All Fields]) OR "gastrointestinal tract"[All Fields] OR ("alimentary"[All Fields] AND "tract"[All Fields]) OR "alimentary tract"[All Fields]) AND ("flora"[All Fields] OR "florae"[All Fields] OR "floras"[All Fields])) OR (("bowel s"[All Fields] OR "bowell"[All Fields] OR "intestines"[MeSH Terms] OR "intestines"[All Fields] OR "bowel"[All Fields] OR "bowels"[All Fields]) AND ("flora"[All Fields] OR "florae"[All Fields] OR "floras"[All Fields])) OR (("gastrointestinal tract"[MeSH Terms] OR ("gastrointestinal"[All Fields] AND "tract"[All Fields]) OR "gastrointestinal tract"[All Fields] OR ("digestive"[All Fields] AND "canal"[All Fields]) OR "digestive canal"[All Fields]) AND ("flora"[All Fields] OR "florae"[All Fields] OR "floras"[All Fields])) OR (("gastrointestinal tract"[MeSH Terms] OR ("gastrointestinal"[All Fields] AND "tract"[All Fields]) OR "gastrointestinal tract"[All Fields] OR ("digestive"[All Fields] AND "tract"[All Fields]) OR "digestive tract"[All Fields]) AND ("flora"[All Fields] OR "florae"[All Fields] OR "floras"[All Fields])) OR (("enteric"[All Fields] OR "enterically"[All Fields] OR "enterics"[All Fields] OR "enteritis"[MeSH Terms] OR "enteritis"[All Fields] OR "enteritides"[All Fields]) AND ("flora"[All Fields] OR "florae"[All Fields] OR "floras"[All Fields])) OR (("enteric"[All Fields] OR "enterically"[All Fields] OR "enterics"[All Fields] OR "enteritis"[MeSH Terms] OR "enteritis"[All Fields] OR "enteritides"[All Fields]) AND ("microbiota"[MeSH Terms] OR "microbiota"[All Fields] OR "microbiotas"[All Fields] OR "microbiota s"[All Fields] OR "microbiotae"[All Fields])) OR (("flora"[All Fields] OR "florae"[All Fields] OR "floras"[All Fields]) AND ("intestinalization"[All Fields] OR "intestinalized"[All Fields] OR "intestinally"[All Fields] OR "intestinals"[All Fields] OR "intestine s"[All Fields] OR "intestines"[MeSH Terms] OR "intestines"[All Fields] OR "intestinal"[All Fields] OR "intestine"[All Fields])) OR (("stomach"[MeSH Terms] OR "stomach"[All Fields] OR "gastro"[All Fields]) AND ("gastrointestinal microbiome"[MeSH Terms] OR ("gastrointestinal"[All Fields] AND "microbiome"[All Fields]) OR "gastrointestinal microbiome"[All Fields] OR ("intestinal"[All Fields] AND "flora"[All Fields]) OR "intestinal flora"[All Fields])) OR (("gastrointestinal"[All Fields] OR "gastrointestinally"[All Fields] OR "gastrointestine"[All Fields]) AND ("canal s"[All Fields] OR "canaled"[All Fields] OR "canals"[All Fields] OR "dental pulp cavity"[MeSH Terms] OR ("dental"[All Fields] AND "pulp"[All Fields] AND "cavity"[All Fields]) OR "dental pulp cavity"[All Fields] OR "canal"[All Fields]) AND ("flora"[All Fields] OR "florae"[All Fields] OR "floras"[All Fields])) OR ("gastrointestinal microbiome"[MeSH Terms] OR ("gastrointestinal"[All Fields] AND "microbiome"[All Fields]) OR "gastrointestinal microbiome"[All Fields] OR ("gastrointestinal"[All Fields] AND "flora"[All Fields]) OR "gastrointestinal flora"[All Fields]) OR ("gastrointestinal microbiome"[MeSH Terms] OR ("gastrointestinal"[All Fields] AND "microbiome"[All Fields]) OR "gastrointestinal microbiome"[All Fields]) OR ("gastrointestinal microbiome"[MeSH Terms] OR ("gastrointestinal"[All Fields] AND "microbiome"[All Fields]) OR "gastrointestinal microbiome"[All Fields] OR ("gastrointestinal"[All Fields] AND "microbiota"[All Fields]) OR "gastrointestinal microbiota"[All Fields]) OR (("gastrointestinal tract"[MeSH Terms] OR ("gastrointestinal"[All Fields] AND "tract"[All Fields]) OR "gastrointestinal tract"[All Fields]) AND ("flora"[All Fields] OR "florae"[All Fields] OR "floras"[All Fields])) OR (("gastrointestinal"[All Fields] OR "gastrointestinally"[All Fields] OR "gastrointestine"[All Fields]) AND ("flora"[All Fields] OR "florae"[All

Fields] OR "floras"[All Fields])) OR (("gastrointestinal"[All Fields] OR "gastrointestinally"[All Fields] OR "gastrointestine"[All Fields]) AND ("tract"[All Fields] OR "tract s"[All Fields] OR "tracts"[All Fields]) AND ("flora"[All Fields] OR "florae"[All Fields] OR "floras"[All Fields])) OR (("gut"[Journal] OR "gut"[All Fields]) AND ("bacteria s"[All Fields] OR "bacteriae"[All Fields] OR "bacterias"[All Fields] OR "microbiology"[MeSH Subheading] OR "microbiology"[All Fields] OR "bacteria"[All Fields] OR "bacteria"[MeSH Terms])) OR ("gastrointestinal microbiome"[MeSH Terms] OR ("gastrointestinal"[All Fields] AND "microbiome"[All Fields]) OR "gastrointestinal microbiome"[All Fields] OR ("gut"[All Fields] AND "microbiota"[All Fields]) OR "gut microbiota"[All Fields]) OR (("intestinalization"[All Fields] OR "intestinalized"[All Fields] OR "intestinally"[All Fields] OR "intestinals"[All Fields] OR "intestine s"[All Fields] OR "intestines"[MeSH Terms] OR "intestines"[All Fields] OR "intestinal"[All Fields] OR "intestine"[All Fields]) AND ("bacteria s"[All Fields] OR "bacteriae"[All Fields] OR "bacterias"[All Fields] OR "microbiology"[MeSH Subheading] OR "microbiology"[All Fields] OR "bacteria"[All Fields] OR "bacteria"[MeSH Terms])) OR (("intestinalization"[All Fields] OR "intestinalized"[All Fields] OR "intestinally"[All Fields] OR "intestinals"[All Fields] OR "intestine s"[All Fields] OR "intestines"[MeSH Terms] OR "intestines"[All Fields] OR "intestinal"[All Fields] OR "intestine"[All Fields]) AND ("bacterial"[All Fields] OR "bacterially"[All Fields] OR "bacterials"[All Fields]) AND ("flora"[All Fields] OR "florae"[All Fields] OR "floras"[All Fields])) OR (("intestinalization"[All Fields] OR "intestinalized"[All Fields] OR "intestinally"[All Fields] OR "intestinals"[All Fields] OR "intestine s"[All Fields] OR "intestines"[MeSH Terms] OR "intestines"[All Fields] OR "intestinal"[All Fields] OR "intestine"[All Fields]) AND ("bacteria"[MeSH Terms] OR "bacteria"[All Fields] OR "bacterium"[All Fields] OR "bacterium s"[All Fields])) OR (("intestinalization"[All Fields] OR "intestinalized"[All Fields] OR "intestinally"[All Fields] OR "intestinals"[All Fields] OR "intestine s"[All Fields] OR "intestines"[MeSH Terms] OR "intestines"[All Fields] OR "intestinal"[All Fields] OR "intestine"[All Fields]) AND ("canal s"[All Fields] OR "canaled"[All Fields] OR "canals"[All Fields] OR "dental pulp cavity"[MeSH Terms] OR ("dental"[All Fields] AND "pulp"[All Fields] AND "cavity"[All Fields]) OR "dental pulp cavity"[All Fields] OR "canal"[All Fields]) AND ("flora"[All Fields] OR "florae"[All Fields] OR "floras"[All Fields])) OR ("gastrointestinal microbiome"[MeSH Terms] OR ("gastrointestinal"[All Fields] AND "microbiome"[All Fields]) OR "gastrointestinal microbiome"[All Fields] OR ("intestinal"[All Fields] AND "flora"[All Fields]) OR "intestinal flora"[All Fields]) OR (("intestinalization"[All Fields] OR "intestinalized"[All Fields] OR "intestinally"[All Fields] OR "intestinals"[All Fields] OR "intestine s"[All Fields] OR "intestines"[MeSH Terms] OR "intestines"[All Fields] OR "intestinal"[All Fields] OR "intestine"[All Fields]) AND ("microbe"[All Fields] OR "microbe s"[All Fields] OR "microbes"[All Fields] OR "microbic"[All Fields])) OR (("intestinalization"[All Fields] OR "intestinalized"[All Fields] OR "intestinally"[All Fields] OR "intestinals"[All Fields] OR "intestine s"[All Fields] OR "intestines"[MeSH Terms] OR "intestines"[All Fields] OR "intestinal"[All Fields] OR "intestine"[All Fields]) AND ("microbe"[All Fields] OR "microbe s"[All Fields] OR "microbes"[All Fields] OR "microbic"[All Fields])) OR ("gastrointestinal microbiome"[MeSH Terms] OR ("gastrointestinal"[All Fields] AND "microbiome"[All Fields]) OR "gastrointestinal microbiome"[All Fields] OR ("intestinal"[All Fields] AND "microbiota"[All Fields]) OR "intestinal microbiota"[All Fields]) OR ("gastrointestinal microbiome"[MeSH Terms] OR ("gastrointestinal"[All Fields] AND "microbiome"[All Fields]) OR "gastrointestinal microbiome"[All Fields] OR ("intestinal"[All Fields] AND "microflora"[All Fields]) OR "intestinal microflora"[All Fields]) OR (("intestinalization"[All Fields] OR "intestinalized"[All Fields] OR "intestinally"[All

Fields] OR "intestinals"[All Fields] OR "intestine s"[All Fields] OR "intestines"[MeSH Terms] OR "intestines"[All Fields] OR "intestinal"[All Fields] OR "intestine"[All Fields]) AND ("microorganism"[All Fields] OR "microorganism s"[All Fields] OR "microorganisms"[All Fields])) OR (("intestines"[MeSH Terms] OR "intestines"[All Fields] OR ("intestinal"[All Fields] AND "tract"[All Fields]) OR "intestinal tract"[All Fields]) AND ("flora"[All Fields] OR "florae"[All Fields] OR "floras"[All Fields])) OR (("intestinalization"[All Fields] OR "intestinalized"[All Fields] OR "intestinally"[All Fields] OR "intestinals"[All Fields] OR "intestine s"[All Fields] OR "intestines"[MeSH Terms] OR "intestines"[All Fields] OR "intestinal"[All Fields] OR "intestine"[All Fields]) AND ("bacteria s"[All Fields] OR "bacteriae"[All Fields] OR "bacterias"[All Fields] OR "microbiology"[MeSH Subheading] OR "microbiology"[All Fields] OR "bacteria"[All Fields] OR "bacteria"[MeSH Terms])) OR (("intestinalization"[All Fields] OR "intestinalized"[All Fields] OR "intestinally"[All Fields] OR "intestinals"[All Fields] OR "intestine s"[All Fields] OR "intestines"[MeSH Terms] OR "intestines"[All Fields] OR "intestinal"[All Fields] OR "intestine"[All Fields]) AND ("bacteria s"[All Fields] OR "bacteriae"[All Fields] OR "bacterias"[All Fields] OR "microbiology"[MeSH Subheading] OR "microbiology"[All Fields] OR "bacteria"[All Fields] OR "bacteria"[MeSH Terms]) AND ("change"[All Fields] OR "changed"[All Fields] OR "changes"[All Fields] OR "changing"[All Fields] OR "changings"[All Fields])) OR (("intestinalization"[All Fields] OR "intestinalized"[All Fields] OR "intestinally"[All Fields] OR "intestinals"[All Fields] OR "intestine s"[All Fields] OR "intestines"[MeSH Terms] OR "intestines"[All Fields] OR "intestinal"[All Fields] OR "intestine"[All Fields]) AND ("bacterial"[All Fields] OR "bacterially"[All Fields] OR "bacterials"[All Fields]) AND ("flora"[All Fields] OR "florae"[All Fields] OR "floras"[All Fields])) OR (("intestinalization"[All Fields] OR "intestinalized"[All Fields] OR "intestinally"[All Fields] OR "intestinals"[All Fields] OR "intestine s"[All Fields] OR "intestines"[MeSH Terms] OR "intestines"[All Fields] OR "intestinal"[All Fields] OR "intestine"[All Fields]) AND ("bacteria"[MeSH Terms] OR "bacteria"[All Fields] OR "bacterium"[All Fields] OR "bacterium s"[All Fields])) OR (("intestinalization"[All Fields] OR "intestinalized"[All Fields] OR "intestinally"[All Fields] OR "intestinals"[All Fields] OR "intestine s"[All Fields] OR "intestines"[MeSH Terms] OR "intestines"[All Fields] OR "intestinal"[All Fields] OR "intestine"[All Fields]) AND ("microbial"[All Fields] OR "microbially"[All Fields] OR "microbials"[All Fields]) AND ("flora"[All Fields] OR "florae"[All Fields] OR "floras"[All Fields])) OR (("intestinalization"[All Fields] OR "intestinalized"[All Fields] OR "intestinally"[All Fields] OR "intestinals"[All Fields] OR "intestine s"[All Fields] OR "intestines"[MeSH Terms] OR "intestines"[All Fields] OR "intestinal"[All Fields] OR "intestine"[All Fields]) AND ("microflora"[All Fields] OR "microflorae"[All Fields] OR "microfloras"[All Fields])) OR (("intestinalization"[All Fields] OR "intestinalized"[All Fields] OR "intestinally"[All Fields] OR "intestinals"[All Fields] OR "intestine s"[All Fields] OR "intestines"[MeSH Terms] OR "intestines"[All Fields] OR "intestinal"[All Fields] OR "intestine"[All Fields]) AND ("flora"[All Fields] OR "florae"[All Fields] OR "floras"[All Fields])) OR ("host"[All Fields] AND "micro-organism"[All Fields] AND ("interact"[All Fields] OR "interactant"[All Fields] OR "interactants"[All Fields] OR "interacted"[All Fields] OR "interacting"[All Fields] OR "interaction"[All Fields] OR "interactional"[All Fields] OR "interactions"[All Fields] OR "interactive"[All Fields] OR "interactively"[All Fields] OR "interactives"[All Fields] OR "interactivities"[All Fields] OR "interactivity"[All Fields] OR "interacts"[All Fields])) OR ("host microbial interactions"[MeSH Terms] OR ("host"[All Fields] AND "microbial"[All Fields] AND "interactions"[All Fields]) OR "host microbial interactions"[All Fields] OR ("host"[All Fields] AND "microbial"[All Fields] AND "interaction"[All

Fields]) OR "host microbial interaction"[All Fields]) OR ("host microbial interactions"[MeSH Terms] OR ("host"[All Fields] AND "microbial"[All Fields] AND "interactions"[All Fields]) OR "host microbial interactions"[All Fields]) OR ("host"[All Fields] AND ("microbiome s"[All Fields] OR "microbiomic"[All Fields] OR "microbiomics"[All Fields] OR "microbiota"[MeSH Terms] OR "microbiota"[All Fields] OR "microbiome"[All Fields] OR "microbiomes"[All Fields]) AND ("interact"[All Fields] OR "interactant"[All Fields] OR "interactants"[All Fields] OR "interacted"[All Fields] OR "interacting"[All Fields] OR "interaction"[All Fields] OR "interactional"[All Fields] OR "interactions"[All Fields] OR "interactive"[All Fields] OR "interactively"[All Fields] OR "interactives"[All Fields] OR "interactivities"[All Fields] OR "interactivity"[All Fields] OR "interacts"[All Fields])) OR ("host microbial interactions"[MeSH Terms] OR ("host"[All Fields] AND "microbial"[All Fields] AND "interactions"[All Fields]) OR "host microbial interactions"[All Fields] OR ("host"[All Fields] AND "microbiota"[All Fields] AND "interaction"[All Fields]) OR "host microbiota interaction"[All Fields]) OR ("host"[All Fields] AND "microbiotal"[All Fields] AND ("interact"[All Fields] OR "interactant"[All Fields] OR "interactants"[All Fields] OR "interacted"[All Fields] OR "interacting"[All Fields] OR "interaction"[All Fields] OR "interactional"[All Fields] OR "interactions"[All Fields] OR "interactive"[All Fields] OR "interactively"[All Fields] OR "interactives"[All Fields] OR "interactivities"[All Fields] OR "interactivity"[All Fields] OR "interacts"[All Fields])) OR ("host"[All Fields] AND ("microbial interactions"[MeSH Terms] OR ("microbial"[All Fields] AND "interactions"[All Fields]) OR "microbial interactions"[All Fields] OR ("microorganism"[All Fields] AND "interaction"[All Fields]) OR "microorganism interaction"[All Fields])) OR ("host microbial interactions"[MeSH Terms] OR ("host"[All Fields] AND "microbial"[All Fields] AND "interactions"[All Fields]) OR "host microbial interactions"[All Fields] OR ("microbe"[All Fields] AND "host"[All Fields] AND "interaction"[All Fields]) OR "microbe host interaction"[All Fields]) OR ("host microbial interactions"[MeSH Terms] OR ("host"[All Fields] AND "microbial"[All Fields] AND "interactions"[All Fields]) OR "host microbial interactions"[All Fields] OR ("microbial"[All Fields] AND "host"[All Fields] AND "interaction"[All Fields]) OR "microbial host interaction"[All Fields]) OR (("microbiome s"[All Fields] OR "microbiomic"[All Fields] OR "microbiomics"[All Fields] OR "microbiota"[MeSH Terms] OR "microbiota"[All Fields] OR "microbiome"[All Fields] OR "microbiomes"[All Fields]) AND "host"[All Fields] AND ("interact"[All Fields] OR "interactant"[All Fields] OR "interactants"[All Fields] OR "interacted"[All Fields] OR "interacting"[All Fields] OR "interaction"[All Fields] OR "interactional"[All Fields] OR "interactions"[All Fields] OR "interactive"[All Fields] OR "interactively"[All Fields] OR "interactives"[All Fields] OR "interactivities"[All Fields] OR "interactivity"[All Fields] OR "interacts"[All Fields])) OR ("host microbial interactions"[MeSH Terms] OR ("host"[All Fields] AND "microbial"[All Fields] AND "interactions"[All Fields]) OR "host microbial interactions"[All Fields] OR ("microbiota"[All Fields] AND "host"[All Fields] AND "interaction"[All Fields]) OR "microbiota host interaction"[All Fields]) OR (("microorganism"[All Fields] OR "microorganism s"[All Fields] OR "microorganisms"[All Fields]) AND "host"[All Fields] AND ("interact"[All Fields] OR "interactant"[All Fields] OR "interactants"[All Fields] OR "interacted"[All Fields] OR "interacting"[All Fields] OR "interaction"[All Fields] OR "interactional"[All Fields] OR "interactions"[All Fields] OR "interactive"[All Fields] OR "interactively"[All Fields] OR "interactives"[All Fields] OR "interactivities"[All Fields] OR "interactivity"[All Fields] OR "interacts"[All Fields])) OR ("host microbial interactions"[MeSH Terms] OR ("host"[All Fields] AND "microbial"[All Fields] AND "interactions"[All Fields]) OR "host microbial interactions"[All Fields] OR ("host"[All

Fields] AND "microbe"[All Fields] AND "interaction"[All Fields]) OR "host microbe interaction"[All Fields]) OR (("gingiva"[MeSH Terms] OR "gingiva"[All Fields] OR "gingivae"[All Fields]) AND ("microflora"[All Fields] OR "microflorae"[All Fields] OR "microfloras"[All Fields])) OR (("mouth"[MeSH Terms] OR "mouth"[All Fields] OR "mouths"[All Fields] OR "mouth s"[All Fields] OR "mouthed"[All Fields] OR "mouthful"[All Fields] OR "mouthfuls"[All Fields] OR "mouthing"[All Fields]) AND ("bacteria s"[All Fields] OR "bacteriae"[All Fields] OR "bacterias"[All Fields] OR "microbiology"[MeSH Subheading] OR "microbiology"[All Fields] OR "bacteria"[All Fields] OR "bacteria"[MeSH Terms])) OR (("mouth"[MeSH Terms] OR "mouth"[All Fields] OR "mouths"[All Fields] OR "mouth s"[All Fields] OR "mouthed"[All Fields] OR "mouthful"[All Fields] OR "mouthfuls"[All Fields] OR "mouthing"[All Fields]) AND ("bacterial"[All Fields] OR "bacterially"[All Fields] OR "bacterials"[All Fields]) AND ("flora"[All Fields] OR "florae"[All Fields] OR "floras"[All Fields])) OR (("mouth"[MeSH Terms] OR "mouth"[All Fields] OR "mouths"[All Fields] OR "mouth s"[All Fields] OR "mouthed"[All Fields] OR "mouthful"[All Fields] OR "mouthfuls"[All Fields] OR "mouthing"[All Fields]) AND ("bacteria"[MeSH Terms] OR "bacteria"[All Fields] OR "bacterium"[All Fields] OR "bacterium s"[All Fields])) OR (("mouth"[MeSH Terms] OR "mouth"[All Fields] OR "mouths"[All Fields] OR "mouth s"[All Fields] OR "mouthed"[All Fields] OR "mouthful"[All Fields] OR "mouthfuls"[All Fields] OR "mouthing"[All Fields]) AND ("microflora"[All Fields] OR "microflorae"[All Fields] OR "microfloras"[All Fields])) OR (("mouth"[MeSH Terms] OR "mouth"[All Fields] OR "oral"[All Fields]) AND ("bacteria s"[All Fields] OR "bacteriae"[All Fields] OR "bacterias"[All Fields] OR "microbiology"[MeSH Subheading] OR "microbiology"[All Fields] OR "bacteria"[All Fields] OR "bacteria"[MeSH Terms])) OR (("mouth"[MeSH Terms] OR "mouth"[All Fields] OR "oral"[All Fields]) AND ("bacterial"[All Fields] OR "bacterially"[All Fields] OR "bacterials"[All Fields]) AND ("flora"[All Fields] OR "florae"[All Fields] OR "floras"[All Fields])) OR (("mouth"[MeSH Terms] OR "mouth"[All Fields] OR "oral"[All Fields]) AND ("bacteria"[MeSH Terms] OR "bacteria"[All Fields] OR "bacterium"[All Fields] OR "bacterium s"[All Fields])) OR (("mouth"[MeSH Terms] OR "mouth"[All Fields] OR ("oral"[All Fields] AND "cavity"[All Fields]) OR "oral cavity"[All Fields]) AND ("flora"[All Fields] OR "florae"[All Fields] OR "floras"[All Fields])) OR (("mouth"[MeSH Terms] OR "mouth"[All Fields] OR "oral"[All Fields]) AND ("flora"[All Fields] OR "florae"[All Fields] OR "floras"[All Fields])) OR (("mouth"[MeSH Terms] OR "mouth"[All Fields] OR "oral"[All Fields]) AND ("microbe"[All Fields] OR "microbe s"[All Fields] OR "microbes"[All Fields] OR "microbic"[All Fields])) OR (("mouth"[MeSH Terms] OR "mouth"[All Fields] OR "oral"[All Fields]) AND ("microbe"[All Fields] OR "microbe s"[All Fields] OR "microbes"[All Fields] OR "microbic"[All Fields])) OR (("mouth"[MeSH Terms] OR "mouth"[All Fields] OR "oral"[All Fields]) AND ("microbial"[All Fields] OR "microbially"[All Fields] OR "microbials"[All Fields]) AND ("flora"[All Fields] OR "florae"[All Fields] OR "floras"[All Fields])) OR (("mouth"[MeSH Terms] OR "mouth"[All Fields] OR "oral"[All Fields]) AND ("microbiota"[MeSH Terms] OR "microbiota"[All Fields] OR "microbiotas"[All Fields] OR "microbiota s"[All Fields] OR "microbiotae"[All Fields])) OR (("mouth"[MeSH Terms] OR "mouth"[All Fields] OR "oral"[All Fields]) AND ("microflora"[All Fields] OR "microflorae"[All Fields] OR "microfloras"[All Fields])) OR (("mouth"[MeSH Terms] OR "mouth"[All Fields] OR "oral"[All Fields]) AND ("microorganism"[All Fields] OR "microorganism s"[All Fields] OR "microorganisms"[All Fields])) OR (("mouth"[MeSH Terms] OR "mouth"[All Fields] OR "oral"[All Fields]) AND ("microorganism"[All Fields] OR "microorganism s"[All Fields] OR "microorganisms"[All Fields])) OR (("pharynx"[MeSH Terms] OR "pharynx"[All Fields] OR

"throat"[All Fields] OR "throats"[All Fields]) AND ("flora"[All Fields] OR "florae"[All Fields] OR "floras"[All Fields])) OR (("mouth"[MeSH Terms] OR "mouth"[All Fields] OR "mouths"[All Fields] OR "mouth s"[All Fields] OR "mouthed"[All Fields] OR "mouthful"[All Fields] OR "mouthfuls"[All Fields] OR "mouthing"[All Fields]) AND ("flora"[All Fields] OR "florae"[All Fields] OR "floras"[All Fields])) OR (("lung"[MeSH Terms] OR "lung"[All Fields]) AND ("flora"[All Fields] OR "florae"[All Fields] OR "floras"[All Fields])) OR (("lung"[MeSH Terms] OR "lung"[All Fields]) AND ("microbiota"[MeSH Terms] OR "microbiota"[All Fields] OR ("microbial"[All Fields] AND "community"[All Fields]) OR "microbial community"[All Fields])) OR (("lung"[MeSH Terms] OR "lung"[All Fields]) AND ("microbiome s"[All Fields] OR "microbiomic"[All Fields] OR "microbiomics"[All Fields] OR "microbiota"[MeSH Terms] OR "microbiota"[All Fields] OR "microbiome"[All Fields] OR "microbiomes"[All Fields])) OR (("lung"[MeSH Terms] OR "lung"[All Fields]) AND ("microflora"[All Fields] OR "microflorae"[All Fields] OR "microfloras"[All Fields])) OR (("lung"[MeSH Terms] OR "lung"[All Fields]) AND ("microorganism"[All Fields] OR "microorganism s"[All Fields] OR "microorganisms"[All Fields])) OR (("lung"[MeSH Terms] OR "lung"[All Fields] OR "pulmonary"[All Fields]) AND ("flora"[All Fields] OR "florae"[All Fields] OR "floras"[All Fields])) OR (("lung"[MeSH Terms] OR "lung"[All Fields] OR "pulmonary"[All Fields]) AND "micro-organisms"[All Fields]) OR (("lung"[MeSH Terms] OR "lung"[All Fields] OR "pulmonary"[All Fields]) AND ("microbiota"[MeSH Terms] OR "microbiota"[All Fields] OR ("microbial"[All Fields] AND "community"[All Fields]) OR "microbial community"[All Fields])) OR (("lung"[MeSH Terms] OR "lung"[All Fields] OR "pulmonary"[All Fields]) AND ("microbiome s"[All Fields] OR "microbiomic"[All Fields] OR "microbiomics"[All Fields] OR "microbiota"[MeSH Terms] OR "microbiota"[All Fields] OR "microbiome"[All Fields] OR "microbiomes"[All Fields])) OR (("lung"[MeSH Terms] OR "lung"[All Fields] OR "pulmonary"[All Fields]) AND ("microbiota"[MeSH Terms] OR "microbiota"[All Fields] OR "microbiotas"[All Fields] OR "microbiota s"[All Fields] OR "microbiotae"[All Fields])) OR (("lung"[MeSH Terms] OR "lung"[All Fields] OR "pulmonary"[All Fields]) AND ("microflora"[All Fields] OR "microflorae"[All Fields] OR "microfloras"[All Fields])) OR (("lung"[MeSH Terms] OR "lung"[All Fields] OR "pulmonary"[All Fields]) AND ("microorganism"[All Fields] OR "microorganism s"[All Fields] OR "microorganisms"[All Fields])) OR (("lung"[MeSH Terms] OR "lung"[All Fields]) AND ("microbiota"[MeSH Terms] OR "microbiota"[All Fields] OR "microbiotas"[All Fields] OR "microbiota s"[All Fields] OR "microbiotae"[All Fields])) OR ("inter-microbial"[All Fields] AND ("interact"[All Fields] OR "interactant"[All Fields] OR "interactants"[All Fields] OR "interacted"[All Fields] OR "interacting"[All Fields] OR "interaction"[All Fields] OR "interactional"[All Fields] OR "interactions"[All Fields] OR "interactive"[All Fields] OR "interactively"[All Fields] OR "interactives"[All Fields] OR "interactivities"[All Fields] OR "interactivity"[All Fields] OR "interacts"[All Fields])) OR ("intermicrobial"[All Fields] AND ("interact"[All Fields] OR "interactant"[All Fields] OR "interactants"[All Fields] OR "interacted"[All Fields] OR "interacting"[All Fields] OR "interaction"[All Fields] OR "interactional"[All Fields] OR "interactions"[All Fields] OR "interactive"[All Fields] OR "interactively"[All Fields] OR "interactives"[All Fields] OR "interactivities"[All Fields] OR "interactivity"[All Fields] OR "interacts"[All Fields])) OR ("microbe-microbe"[All Fields] AND ("associate"[All Fields] OR "associated"[All Fields] OR "associates"[All Fields] OR "associating"[All Fields] OR "association"[MeSH Terms] OR "association"[All Fields] OR "associations"[All Fields])) OR ("microbe-microbe"[All Fields] AND ("interact"[All Fields] OR "interactant"[All Fields] OR "interactants"[All Fields] OR "interacted"[All Fields] OR "interacting"[All Fields]

OR "interaction"[All Fields] OR "interactional"[All Fields] OR "interactions"[All Fields] OR "interactive"[All Fields] OR "interactively"[All Fields] OR "interactives"[All Fields] OR "interactivities"[All Fields] OR "interactivity"[All Fields] OR "interacts"[All Fields])) OR ("microbe-microbe"[All Fields] AND ("relationship"[All Fields] OR "relationships"[All Fields])) OR ("microbe-microbe"[All Fields] AND ("system"[All Fields] OR "system s"[All Fields] OR "systems"[All Fields])) OR ("microbial-microbial"[All Fields] AND ("interact"[All Fields] OR "interactant"[All Fields] OR "interactants"[All Fields] OR "interacted"[All Fields] OR "interacting"[All Fields] OR "interaction"[All Fields] OR "interactional"[All Fields] OR "interactions"[All Fields] OR "interactive"[All Fields] OR "interactively"[All Fields] OR "interactives"[All Fields] OR "interactivities"[All Fields] OR "interactivity"[All Fields] OR "interacts"[All Fields])) OR ("microbiota microbiome"[All Fields] AND ("interact"[All Fields] OR "interactant"[All Fields] OR "interactants"[All Fields] OR "interacted"[All Fields] OR "interacting"[All Fields] OR "interaction"[All Fields] OR "interactional"[All Fields] OR "interactions"[All Fields] OR "interactive"[All Fields] OR "interactively"[All Fields] OR "interactives"[All Fields] OR "interactivities"[All Fields] OR "interactivity"[All Fields] OR "interacts"[All Fields])) OR (("microorganism"[All Fields] OR "microorganism s"[All Fields] OR "microorganisms"[All Fields]) AND ("interrelate"[All Fields] OR "interrelated"[All Fields] OR "interrelates"[All Fields] OR "interrelating"[All Fields] OR "interrelation"[All Fields] OR "interrelations"[All Fields])) OR ("microorganism-microorganism"[All Fields] AND ("interact"[All Fields] OR "interactant"[All Fields] OR "interactants"[All Fields] OR "interacted"[All Fields] OR "interacting"[All Fields] OR "interaction"[All Fields] OR "interactional"[All Fields] OR "interactions"[All Fields] OR "interactive"[All Fields] OR "interactively"[All Fields] OR "interactives"[All Fields] OR "interactivities"[All Fields] OR "interactivity"[All Fields] OR "interacts"[All Fields])) OR ("microbe-microbe"[All Fields] AND ("interact"[All Fields] OR "interactant"[All Fields] OR "interactants"[All Fields] OR "interacted"[All Fields] OR "interacting"[All Fields] OR "interaction"[All Fields] OR "interactional"[All Fields] OR "interactions"[All Fields] OR "interactive"[All Fields] OR "interactively"[All Fields] OR "interactives"[All Fields] OR "interactivities"[All Fields] OR "interactivity"[All Fields] OR "interacts"[All Fields])) OR ("dysbiosis"[MeSH Terms] OR "dysbiosis"[All Fields] OR "dysbioses"[All Fields]) OR ("dysbiosis"[MeSH Terms] OR "dysbiosis"[All Fields] OR "dysbioses"[All Fields]) OR ("dysbiosis"[MeSH Terms] OR "dysbiosis"[All Fields] OR "disbiosis"[All Fields]) OR ("dysbiosis"[MeSH Terms] OR "dysbiosis"[All Fields] OR "disbioses"[All Fields]) OR ("dysbiosis"[MeSH Terms] OR "dysbiosis"[All Fields] OR ("dys"[All Fields] AND "symbiosis"[All Fields])) OR ("dysbiosis"[MeSH Terms] OR "dysbiosis"[All Fields] OR ("dys"[All Fields] AND "symbiosis"[All Fields])) OR ("dysbiosis"[MeSH Terms] OR "dysbiosis"[All Fields] OR ("dys"[All Fields] AND "symbioses"[All Fields])) OR ("dysbiosis"[MeSH Terms] OR "dysbiosis"[All Fields] OR "dysbacteriosis"[All Fields]) OR ("dysbiosis"[MeSH Terms] OR "dysbiosis"[All Fields] OR "dysbacterioses"[All Fields]) OR ("dysbiosis"[MeSH Terms] OR "dysbiosis"[All Fields] OR "disbacteriosis"[All Fields]) OR ("dysbiosis"[MeSH Terms] OR "dysbiosis"[All Fields]) OR ("gastrointestinal microbiome"[MeSH Terms] OR ("gastrointestinal"[All Fields] AND "microbiome"[All Fields]) OR "gastrointestinal microbiome"[All Fields] OR ("gastrointestinal"[All Fields] AND "microbiomes"[All Fields]) OR "gastrointestinal microbiomes"[All Fields]) OR ("gastrointestinal microbiome"[MeSH Terms] OR ("gastrointestinal"[All Fields] AND "microbiome"[All Fields]) OR "gastrointestinal microbiome"[All Fields] OR ("microbiome"[All Fields] AND "gastrointestinal"[All Fields]) OR "microbiome gastrointestinal"[All Fields]) OR ("gastrointestinal mi-

crobiome"[MeSH Terms] OR ("gastrointestinal"[All Fields] AND "microbiome"[All Fields]) OR "gastrointestinal microbiome"[All Fields] OR ("gut"[All Fields] AND "microbiome"[All Fields]) OR "gut microbiome"[All Fields]) OR ("gastrointestinal microbiome"[MeSH Terms] OR ("gastrointestinal"[All Fields] AND "microbiome"[All Fields]) OR "gastrointestinal microbiome"[All Fields] OR ("gut"[All Fields] AND "microbiomes"[All Fields]) OR "gut microbiomes"[All Fields]) OR ("gastrointestinal microbiome"[MeSH Terms] OR ("gastrointestinal"[All Fields] AND "microbiome"[All Fields]) OR "gastrointestinal microbiome"[All Fields] OR ("microbiome"[All Fields] AND "gut"[All Fields]) OR "microbiome gut"[All Fields]) OR ("gastrointestinal microbiome"[MeSH Terms] OR ("gastrointestinal"[All Fields] AND "microbiome"[All Fields]) OR "gastrointestinal microbiome"[All Fields] OR ("gut"[All Fields] AND "microflora"[All Fields]) OR "gut microflora"[All Fields]) OR ("gastrointestinal microbiome"[MeSH Terms] OR ("gastrointestinal"[All Fields] AND "microbiome"[All Fields]) OR "gastrointestinal microbiome"[All Fields] OR ("microflora"[All Fields] AND "gut"[All Fields]) OR "microflora gut"[All Fields]) OR ("gastrointestinal microbiome"[MeSH Terms] OR ("gastrointestinal"[All Fields] AND "microbiome"[All Fields]) OR "gastrointestinal microbiome"[All Fields] OR ("gut"[All Fields] AND "microbiotas"[All Fields]) OR "gut microbiotas"[All Fields]) OR ("gastrointestinal microbiome"[MeSH Terms] OR ("gastrointestinal"[All Fields] AND "microbiome"[All Fields]) OR "gastrointestinal microbiome"[All Fields] OR ("microbiota"[All Fields] AND "gut"[All Fields]) OR "microbiota gut"[All Fields]) OR ("gastrointestinal microbiome"[MeSH Terms] OR ("gastrointestinal"[All Fields] AND "microbiome"[All Fields]) OR "gastrointestinal microbiome"[All Fields] OR ("flora"[All Fields] AND "gastrointestinal"[All Fields]) OR "flora gastrointestinal"[All Fields]) OR ("gastrointestinal microbiome"[MeSH Terms] OR ("gastrointestinal"[All Fields] AND "microbiome"[All Fields]) OR "gastrointestinal microbiome"[All Fields] OR ("gut"[All Fields] AND "flora"[All Fields]) OR "gut flora"[All Fields]) OR ("gastrointestinal microbiome"[MeSH Terms] OR ("gastrointestinal"[All Fields] AND "microbiome"[All Fields]) OR "gastrointestinal microbiome"[All Fields] OR ("flora"[All Fields] AND "gut"[All Fields]) OR "flora gut"[All Fields]) OR ("gastrointestinal microbiome"[MeSH Terms] OR ("gastrointestinal"[All Fields] AND "microbiome"[All Fields]) OR "gastrointestinal microbiome"[All Fields] OR ("gastrointestinal"[All Fields] AND "microbiotas"[All Fields]) OR "gastrointestinal microbiotas"[All Fields]) OR ("gastrointestinal microbiome"[MeSH Terms] OR ("gastrointestinal"[All Fields] AND "microbiome"[All Fields]) OR "gastrointestinal microbiome"[All Fields] OR ("microbiota"[All Fields] AND "gastrointestinal"[All Fields]) OR "microbiota gastrointestinal"[All Fields]) OR ("gastrointestinal microbiome"[MeSH Terms] OR ("gastrointestinal"[All Fields] AND "microbiome"[All Fields]) OR "gastrointestinal microbiome"[All Fields] OR ("gastrointestinal"[All Fields] AND "microbial"[All Fields] AND "community"[All Fields]) OR "gastrointestinal microbial community"[All Fields]) OR ("gastrointestinal microbiome"[MeSH Terms] OR ("gastrointestinal"[All Fields] AND "microbiome"[All Fields]) OR "gastrointestinal microbiome"[All Fields] OR ("gastrointestinal"[All Fields] AND "microbial"[All Fields] AND "communities"[All Fields]) OR "gastrointestinal microbial communities"[All Fields]) OR ("gastrointestinal microbiome"[MeSH Terms] OR ("gastrointestinal"[All Fields] AND "microbiome"[All Fields]) OR "gastrointestinal microbiome"[All Fields] OR ("microbial"[All Fields] AND "community"[All Fields] AND "gastrointestinal"[All Fields])) OR ("gastrointestinal microbiome"[MeSH Terms] OR ("gastrointestinal"[All Fields] AND "microbiome"[All Fields]) OR "gastrointestinal microbiome"[All Fields] OR ("gastrointestinal"[All Fields] AND "microflora"[All Fields])

OR "gastrointestinal microflora"[All Fields]) OR ("gastrointestinal microbiome"[MeSH Terms] OR ("gastrointestinal"[All Fields] AND "microbiome"[All Fields]) OR "gastrointestinal microbiome"[All Fields] OR ("microflora"[All Fields] AND "gastrointestinal"[All Fields]) OR "microflora gastrointestinal"[All Fields]) OR ("gastrointestinal microbiome"[MeSH Terms] OR ("gastrointestinal"[All Fields] AND "microbiome"[All Fields]) OR "gastrointestinal microbiome"[All Fields] OR ("gastric"[All Fields] AND "microbiome"[All Fields]) OR "gastric microbiome"[All Fields]) OR ("gastrointestinal microbiome"[MeSH Terms] OR ("gastrointestinal"[All Fields] AND "microbiome"[All Fields]) OR "gastrointestinal microbiome"[All Fields] OR ("gastric"[All Fields] AND "microbiomes"[All Fields]) OR "gastric microbiomes"[All Fields]) OR ("gastrointestinal microbiome"[MeSH Terms] OR ("gastrointestinal"[All Fields] AND "microbiome"[All Fields]) OR "gastrointestinal microbiome"[All Fields] OR ("microbiome"[All Fields] AND "gastric"[All Fields]) OR "microbiome gastric"[All Fields]) OR ("gastrointestinal microbiome"[MeSH Terms] OR ("gastrointestinal"[All Fields] AND "microbiome"[All Fields]) OR "gastrointestinal microbiome"[All Fields] OR ("intestinal"[All Fields] AND "microbiome"[All Fields]) OR "intestinal microbiome"[All Fields]) OR ("gastrointestinal microbiome"[MeSH Terms] OR ("gastrointestinal"[All Fields] AND "microbiome"[All Fields]) OR "gastrointestinal microbiome"[All Fields] OR ("intestinal"[All Fields] AND "microbiomes"[All Fields]) OR "intestinal microbiomes"[All Fields]) OR ("gastrointestinal microbiome"[MeSH Terms] OR ("gastrointestinal"[All Fields] AND "microbiome"[All Fields]) OR "gastrointestinal microbiome"[All Fields] OR ("microbiome"[All Fields] AND "intestinal"[All Fields]) OR "microbiome intestinal"[All Fields]) OR ("gastrointestinal microbiome"[MeSH Terms] OR ("gastrointestinal"[All Fields] AND "microbiome"[All Fields]) OR "gastrointestinal microbiome"[All Fields] OR ("intestinal"[All Fields] AND "microbiotas"[All Fields]) OR "intestinal microbiotas"[All Fields]) OR ("gastrointestinal microbiome"[MeSH Terms] OR ("gastrointestinal"[All Fields] AND "microbiome"[All Fields]) OR "gastrointestinal microbiome"[All Fields] OR ("microbiota"[All Fields] AND "intestinal"[All Fields]) OR "microbiota intestinal"[All Fields]) OR ("gastrointestinal microbiome"[MeSH Terms] OR ("gastrointestinal"[All Fields] AND "microbiome"[All Fields]) OR "gastrointestinal microbiome"[All Fields] OR ("microflora"[All Fields] AND "intestinal"[All Fields]) OR "microflora intestinal"[All Fields]) OR ("gastrointestinal microbiome"[MeSH Terms] OR ("gastrointestinal"[All Fields] AND "microbiome"[All Fields]) OR "gastrointestinal microbiome"[All Fields] OR ("flora"[All Fields] AND "intestinal"[All Fields]) OR "flora intestinal"[All Fields]) OR ("gastrointestinal microbiome"[MeSH Terms] OR ("gastrointestinal"[All Fields] AND "microbiome"[All Fields]) OR "gastrointestinal microbiome"[All Fields] OR ("enteric"[All Fields] AND "bacteria"[All Fields]) OR "enteric bacteria"[All Fields]) OR ("gastrointestinal microbiome"[MeSH Terms] OR ("gastrointestinal"[All Fields] AND "microbiome"[All Fields]) OR "gastrointestinal microbiome"[All Fields] OR ("bacteria"[All Fields] AND "enteric"[All Fields]) OR "bacteria enteric"[All Fields]) OR ("virome"[MeSH Terms] OR "virome"[All Fields] OR "viromes"[All Fields] OR "viromic"[All Fields] OR "viromics"[All Fields]) OR ("virome"[MeSH Terms] OR "virome"[All Fields] OR ("virus"[All Fields] AND "microbiome"[All Fields]) OR "virus microbiome"[All Fields]) OR ("virome"[MeSH Terms] OR "virome"[All Fields] OR ("microbiome"[All Fields] AND "virus"[All Fields]) OR "microbiome virus"[All Fields]) OR ("virome"[MeSH Terms] OR "virome"[All Fields] OR ("microbiomes"[All Fields] AND "virus"[All Fields])) OR ("virome"[MeSH Terms] OR "virome"[All Fields] OR ("virus"[All Fields] AND "microbiomes"[All Fields])) OR ("virome"[MeSH Terms] OR "virome"[All Fields] OR ("viral"[All Fields] AND

	"microbiome"[All Fields]) OR "viral microbiome"[All Fields]) OR ("virome"[MeSH Terms] OR "virome"[All Fields] OR ("microbiome"[All Fields] AND "viral"[All Fields]) OR "microbiome viral"[All Fields]) OR ("virome"[MeSH Terms] OR "virome"[All Fields] OR ("microbiomes"[All Fields] AND "viral"[All Fields])) OR ("virome"[MeSH Terms] OR "virome"[All Fields] OR ("viral"[All Fields] AND "microbiomes"[All Fields]) OR "viral microbiomes"[All Fields]) OR ("virome"[MeSH Terms] OR "virome"[All Fields] OR "phageome"[All Fields]) OR ("virome"[MeSH Terms] OR "virome"[All Fields] OR "phageomes"[All Fields]) OR (("bacterial"[All Fields] OR "bacterially"[All Fields] OR "bacterials"[All Fields]) AND ("biome s"[All Fields] OR "ecosystem"[MeSH Terms] OR "ecosystem"[All Fields] OR "biome"[All Fields] OR "biomes"[All Fields])) OR "bacteriobiome"[All Fields] OR ("bacteriome"[All Fields] OR "bacteriomes"[All Fields]) OR (("bacterial"[All Fields] OR "bacterially"[All Fields] OR "bacterials"[All Fields]) AND ("microbiome s"[All Fields] OR "microbiomic"[All Fields] OR "microbiomics"[All Fields] OR "microbiota"[MeSH Terms] OR "microbiota"[All Fields] OR "microbiome"[All Fields] OR "microbiomes"[All Fields])) OR "phagome"[All Fields] OR (("virally"[All Fields] OR "virals"[All Fields] OR "virology"[MeSH Terms] OR "virology"[All Fields] OR "viral"[All Fields]) AND ("biome s"[All Fields] OR "ecosystem"[MeSH Terms] OR "ecosystem"[All Fields] OR "biome"[All Fields] OR "biomes"[All Fields])) OR ("virome"[MeSH Terms] OR "virome"[All Fields] OR ("viral"[All Fields] AND "microbiome"[All Fields]) OR "viral microbiome"[All Fields]) OR ("virome"[MeSH Terms] OR "virome"[All Fields] OR ("virus"[All Fields] AND "microbiome"[All Fields]) OR "virus microbiome"[All Fields]) OR ("virome"[MeSH Terms] OR "virome"[All Fields] OR "viromes"[All Fields] OR "viromic"[All Fields] OR "viromics"[All Fields])	
#3	#1 AND #2	238 (Filter 80)
Cochrane Library (Trials)		
#1	Chronic rhinosinusitis with nasal polyps OR nose polyp OR Nasal polyps OR Nasal Polyp OR Polyp, Nasal OR Polyps, Nasal OR nasal cavity polyp OR nasal polyposis OR nose polyposis OR polyp of the nasal cavity OR polyp of the nose OR polyp, nose OR polyposis nasi OR polyposis of the nose OR sino-nasal polyp	1,909 (1,855)
#2	Microbiota OR Microbiotas OR Microbial Community OR Community Microbial OR Microbial Communities OR Microbial Community Composition OR Community Composition Microbial OR Composition Microbial Community OR Microbial Community Compositions OR Microbial Community Structure OR Community Structure Microbial OR Microbial Community Structures OR Microbiome OR Microbiomes OR Human Microbiome OR Human Microbiomes OR Microbiome Human OR microflora OR microbial flora OR bacterial flora OR bacterial microbiota OR bacteriologic flora OR bacterium flora OR neonatal bacterial flora OR bowel microbiota OR alimentary canal flora OR alimentary tract flora OR bowel flora OR digestive canal flora OR digestive tract flora OR enteric flora OR enteric microbiota OR flora intestine OR gastro intestinal flora OR gastrointestinal canal flora OR gastrointestinal flora OR gastrointestinal microbiome OR gastrointestinal microbiota OR gastrointestinal tract flora OR gastrointestine flora OR gastrointestine tract flora OR gut bacteria OR gut microbiota OR intestinal bacteria OR intestinal bacterial flora OR intestinal bacterium OR intestinal canal flora OR intestinal flora OR intestinal microbe OR intestinal microbes OR intestinal microbiota OR intestinal microflora OR intestinal microorganism OR intestinal tract flora OR intestine bacteria OR intestine bacteria change OR intestine bacterial flora OR	22,327 (21,298)

	intestine bacterium OR intestine microbial flora OR intestine microflora OR intestine flora OR host micro-organism interaction OR host microbial interaction OR host microbial interactions OR host microbiome interaction OR host microbiota interaction OR host microbiotal interaction OR host microorganism interaction OR microbe host interaction OR microbial host interaction OR microbiome host interaction OR microbiota host interaction OR microorganism host interaction OR host microbe interaction OR gingiva microflora OR mouth bacteria OR mouth bacterial flora OR mouth bacterium OR mouth microflora OR oral bacteria OR oral bacterial flora OR oral bacterium OR oral cavity flora OR oral flora OR oral microbe OR oral microbes OR oral microbial flora OR oral microbiota OR oral microflora OR oral microorganism OR oral microorganisms OR throat flora OR mouth flora OR lung flora OR lung microbial community OR lung microbiome OR lung microflora OR lung microorganisms OR pulmonary flora OR pulmonary micro-organisms OR pulmonary microbial community OR pulmonary microbiome OR pulmonary microbiota OR pulmonary microflora OR pulmonary micro-organisms OR lung microbiota OR inter-microbial interaction OR intermicrobial interaction OR microbe-microbe associations OR microbe-microbe interactions OR microbe-microbe relationships OR microbe-microbe system OR microbial-microbial interactions OR microbiota interactions OR microbiome interactions OR microorganism interrelation OR microorganism-microorganism interaction OR microbe-microbe interaction OR Dysbiosis OR Dysbioses OR Disbiosis OR Disbioses OR Dys-symbiosis OR Dys symbiosis OR Dys-symbioses OR Dysbacteriosis OR Dysbacterioses OR Disbacteriosis OR Disbacterioses OR Gastrointestinal Microbiomes OR Microbiome Gastrointestinal OR Gut Microbiome OR Gut Microbiomes OR Microbiome Gut OR Gut Microflora OR Microflora Gut OR Gut Microbiotas OR Microbiota Gut OR Flora Gastrointestinal OR Gut Flora OR Flora Gut OR Gastrointestinal Microbiotas OR Microbiota Gastrointestinal OR Gastrointestinal Microbial Community OR Gastrointestinal Microbial Communities OR Microbial Community Gastrointestinal OR Gastrointestinal Microflora OR Microflora Gastrointestinal OR Gastric Microbiome OR Gastric Microbiomes OR Microbiome Gastric OR Intestinal Microbiome OR Intestinal Microbiomes OR Microbiome Intestinal OR Intestinal Microbiotas OR Microbiota Intestinal OR Microflora Intestinal OR Flora Intestinal OR Enteric Bacteria OR Bacteria Enteric OR Viromes OR Virus Microbiome OR Microbiome Virus OR Microbiomes Virus OR Virus Microbiomes OR Viral Microbiome OR Microbiome Viral OR Microbiomes Viral OR Viral Microbiomes OR Phageome OR Phageomes OR bacterial biome OR bacteriobiome OR bacteriome OR bacterial microbiome OR phagome OR viral biome OR viral microbiome OR virus microbiome OR virome	
#3	#1 AND #2	54 (31)
Scopus		
#1	TITLE-ABS-KEY (CRSwNP) OR TITLE-ABS-KEY (Chronic rhinosinusitis with nasal polyps) OR TITLE-ABS-KEY (nose polyp) OR TITLE-ABS-KEY (Nasal polyps) OR TITLE-ABS-KEY (Nasal Polyp) OR TITLE-ABS-KEY (Polyp, Nasal) OR TITLE-ABS-KEY (Polyps, Nasal) OR TITLE-ABS-KEY (nasal cavity polyp) OR TITLE-ABS-KEY (nasal polyposis) OR TITLE-ABS-KEY (nose polyposis) OR TITLE-ABS-KEY (polyp of the nasal cavity) OR TITLE-ABS-KEY (polyp of the nose) OR TITLE-ABS-KEY ("polyp, nose") OR TITLE-ABS-KEY (polyposis nasi) OR TITLE-ABS-KEY (polyposis of the nose) OR TITLE-ABS-KEY (sino-nasal polyp)	17,245
#2	TITLE-ABS-KEY (microbiota) OR TITLE-ABS-KEY (microbiotas) OR TITLE-ABS-KEY (microbial AND community) OR TITLE-ABS-KEY (community, AND	577,910

microbial) OR TITLE-ABS-KEY (microbial AND communities) OR TITLE-ABS-KEY (microbial AND community AND composition) OR TITLE-ABS-KEY (community AND composition, AND microbial) OR TITLE-ABS-KEY (composition, AND microbial AND community) OR TITLE-ABS-KEY (microbial AND community AND compositions) OR TITLE-ABS-KEY (microbial AND community AND structure) OR TITLE-ABS-KEY (community AND structure, AND microbial) OR TITLE-ABS-KEY (microbial AND community AND structures) OR TITLE-ABS-KEY (microbiome) OR TITLE-ABS-KEY (microbiomes) OR TITLE-ABS-KEY (human AND microbiome) OR TITLE-ABS-KEY (human AND microbiomes) OR TITLE-ABS-KEY (microbiome, AND human) OR TITLE-ABS-KEY (microflora) OR TITLE-ABS-KEY (microbial AND flora) OR TITLE-ABS-KEY (bacterial AND flora) OR TITLE-ABS-KEY (bacterial AND microbiota) OR TITLE-ABS-KEY (bacteriologic AND flora) OR TITLE-ABS-KEY (bacterium AND flora) OR TITLE-ABS-KEY (neonatal AND bacterial AND flora) OR TITLE-ABS-KEY (bowel AND microbiota) OR TITLE-ABS-KEY (alimentary AND canal AND flora) OR TITLE-ABS-KEY (alimentary AND tract AND flora) OR TITLE-ABS-KEY (bowel AND flora) OR TITLE-ABS-KEY (digestive AND canal AND flora) OR TITLE-ABS-KEY (digestive AND tract AND flora) OR TITLE-ABS-KEY (enteric AND flora) OR TITLE-ABS-KEY (enteric AND microbiota) OR TITLE-ABS-KEY (flora AND intestine) OR TITLE-ABS-KEY (gastro AND intestinal AND flora) OR TITLE-ABS-KEY (gastrointestinal AND canal AND flora) OR TITLE-ABS-KEY (gastrointestinal AND flora) OR TITLE-ABS-KEY (gastrointestinal AND microbiome) OR TITLE-ABS-KEY (gastrointestinal AND microbiota) OR TITLE-ABS-KEY (gastrointestinal AND tract AND flora) OR TITLE-ABS-KEY (gastrointestine AND flora) OR TITLE-ABS-KEY (gastrointestine AND tract AND flora) OR TITLE-ABS-KEY (gut AND bacteria) OR TITLE-ABS-KEY (gut AND microbiota) OR TITLE-ABS-KEY (intestinal AND bacteria) OR TITLE-ABS-KEY (intestinal AND bacterial AND flora) OR TITLE-ABS-KEY (intestinal AND bacterium) OR TITLE-ABS-KEY (intestinal AND canal AND flora) OR TITLE-ABS-KEY (intestinal AND flora) OR TITLE-ABS-KEY (intestinal AND microbe) OR TITLE-ABS-KEY (intestinal AND microbes) OR TITLE-ABS-KEY (intestinal AND microbiota) OR TITLE-ABS-KEY (intestinal AND microflora) OR TITLE-ABS-KEY (intestinal AND microorganism) OR TITLE-ABS-KEY (intestinal AND tract AND flora) OR TITLE-ABS-KEY (intestine AND bacteria) OR TITLE-ABS-KEY (intestine AND bacteria AND change) OR TITLE-ABS-KEY (intestine AND bacterial AND flora) OR TITLE-ABS-KEY (intestine AND bacterium) OR TITLE-ABS-KEY (intestine AND microbial AND flora) OR TITLE-ABS-KEY (intestine AND microflora) OR TITLE-ABS-KEY (intestine AND flora) OR TITLE-ABS-KEY (host AND micro-organism AND interaction) OR TITLE-ABS-KEY (host AND microbial AND interaction) OR TITLE-ABS-KEY (host AND microbial AND interactions) OR TITLE-ABS-KEY (host AND microbiome AND interaction) OR TITLE-ABS-KEY (host AND microbiota AND interaction) OR TITLE-ABS-KEY (host AND microbiotal AND interaction) OR TITLE-ABS-KEY (host AND microorganism AND interaction) OR TITLE-ABS-KEY (microbe AND host AND interaction) OR TITLE-ABS-KEY (microbial AND host AND interaction) OR TITLE-ABS-KEY (microbiome AND host AND interaction) OR TITLE-ABS-KEY (microbiota AND host AND interaction) OR TITLE-ABS-KEY (microorganism AND host AND interaction) OR TITLE-ABS-KEY (host AND microbe AND interaction) OR TITLE-ABS-KEY (gingiva AND microflora) OR TITLE-ABS-KEY (mouth AND bacteria) OR TITLE-ABS-KEY (mouth AND bacterial AND flora) OR TITLE-ABS-KEY (mouth AND bacterium) OR TITLE-ABS-KEY (mouth AND microflora) OR TITLE-ABS-KEY (oral AND bacteria) OR TITLE-ABS-KEY (oral AND bacterial AND flora) OR TITLE-ABS-

KEY (oral AND bacterium) OR TITLE-ABS-KEY (oral AND cavity AND flora) OR TITLE-ABS-KEY (oral AND flora) OR TITLE-ABS-KEY (oral AND microbe) OR TITLE-ABS-KEY (oral AND microbes) OR TITLE-ABS-KEY (oral AND microbial AND flora) OR TITLE-ABS-KEY (oral AND microbiota) OR TITLE-ABS-KEY (oral AND microflora) OR TITLE-ABS-KEY (oral AND microorganism) OR TITLE-ABS-KEY (oral AND microorganisms) OR TITLE-ABS-KEY (throat AND flora) OR TITLE-ABS-KEY (mouth AND flora) OR TITLE-ABS-KEY (lung AND flora) OR TITLE-ABS-KEY (lung AND microbial AND community) OR TITLE-ABS-KEY (lung AND microbiome) OR TITLE-ABS-KEY (lung AND microflora) OR TITLE-ABS-KEY (lung AND microorganisms) OR TITLE-ABS-KEY (pulmonary AND flora) OR TITLE-ABS-KEY (pulmonary AND micro-organisms) OR TITLE-ABS-KEY (pulmonary AND microbial AND community) OR TITLE-ABS-KEY (pulmonary AND microbiome) OR TITLE-ABS-KEY (pulmonary AND microbiota) OR TITLE-ABS-KEY (pulmonary AND microflora) OR TITLE-ABS-KEY (pulmonary AND microorganisms) OR TITLE-ABS-KEY (lung AND microbiota) OR TITLE-ABS-KEY (inter-microbial AND interaction) OR TITLE-ABS-KEY (microorganisms AND interaction) OR TITLE-ABS-KEY (intermicrobial AND interaction) OR TITLE-ABS-KEY (microbe-microbe AND associations) OR TITLE-ABS-KEY (microbe-microbe AND interactions) OR TITLE-ABS-KEY (microbe-microbe AND relationships) OR TITLE-ABS-KEY (microbe-microbe AND system) OR TITLE-ABS-KEY (microbial-microbial AND interactions) OR TITLE-ABS-KEY (microbiota/microbiome AND interactions) OR TITLE-ABS-KEY (microorganism AND interrelation) OR TITLE-ABS-KEY (microorganism-microorganism AND interaction) OR TITLE-ABS-KEY (microbe-microbe AND interaction) OR TITLE-ABS-KEY (dysbiosis) OR TITLE-ABS-KEY (dysbioses) OR TITLE-ABS-KEY (disbiosis) OR TITLE-ABS-KEY (disbioses) OR TITLE-ABS-KEY (dys-symbiosis) OR TITLE-ABS-KEY (dys AND symbiosis) OR TITLE-ABS-KEY (dys-symbioses) OR TITLE-ABS-KEY (dysbacteriosis) OR TITLE-ABS-KEY (dysbacterioses) OR TITLE-ABS-KEY (disbacteriosis) OR TITLE-ABS-KEY (disbacterioses) OR TITLE-ABS-KEY (gastrointestinal AND microbiomes) OR TITLE-ABS-KEY (microbiome, AND gastrointestinal) OR TITLE-ABS-KEY (gut AND microbiome) OR TITLE-ABS-KEY (gut AND microbiomes) OR TITLE-ABS-KEY (microbiome, AND gut) OR TITLE-ABS-KEY (gut AND microflora) OR TITLE-ABS-KEY (microflora, AND gut) OR TITLE-ABS-KEY (gut AND microbiotas) OR TITLE-ABS-KEY (microbiota, AND gut) OR TITLE-ABS-KEY (flora, AND gastrointestinal) OR TITLE-ABS-KEY (gut AND flora) OR TITLE-ABS-KEY (flora, AND gut) OR TITLE-ABS-KEY (gastrointestinal AND microbiotas) OR TITLE-ABS-KEY (microbiota, AND gastrointestinal) OR TITLE-ABS-KEY (gastrointestinal AND microbial AND community) OR TITLE-ABS-KEY (gastrointestinal AND microbial AND communities) OR TITLE-ABS-KEY (microbial AND community, AND gastrointestinal) OR TITLE-ABS-KEY (gastrointestinal AND microflora) OR TITLE-ABS-KEY (microflora, AND gastrointestinal) OR TITLE-ABS-KEY (gastric AND microbiome) OR TITLE-ABS-KEY (gastric AND microbiomes) OR TITLE-ABS-KEY (microbiome, AND gastric) OR TITLE-ABS-KEY (intestinal AND microbiome) OR TITLE-ABS-KEY (intestinal AND microbiomes) OR TITLE-ABS-KEY (microbiome, AND intestinal) OR TITLE-ABS-KEY (intestinal AND microbiotas) OR TITLE-ABS-KEY (microbiota, AND intestinal) OR TITLE-ABS-KEY (microflora, AND intestinal) OR TITLE-ABS-KEY (flora, AND intestinal) OR TITLE-ABS-KEY (enteric AND bacteria) OR TITLE-ABS-KEY (bacteria, AND enteric) OR TITLE-ABS-KEY (viromes) OR TITLE-ABS-KEY (virus AND microbiome) OR TITLE-ABS-KEY (microbiome, AND virus) OR TITLE-ABS-KEY (microbiomes, AND virus) OR TITLE-ABS-KEY (virus AND microbiomes) OR TITLE-ABS-KEY (viral AND

	microbiome) OR TITLE-ABS-KEY (microbiome, AND viral) OR TITLE-ABS-KEY (microbiomes, AND viral) OR TITLE-ABS-KEY (viral AND microbiomes) OR TITLE-ABS-KEY (phageome) OR TITLE-ABS-KEY (phageomes) OR TITLE-ABS-KEY (bacterial AND biome) OR TITLE-ABS-KEY (bacteriobiome) OR TITLE-ABS-KEY (bacteriome) OR TITLE-ABS-KEY (bacterial AND microbiome) OR TITLE-ABS-KEY (phagome) OR TITLE-ABS-KEY (viral AND biome) OR TITLE-ABS-KEY (viral AND microbiome) OR TITLE-ABS-KEY (virus AND microbiome) OR TITLE-ABS-KEY (virome)	
#3	#1 AND #2	314
**Embase**		
#1	'crswnp' OR 'chronic rhinosinusitis with nasal polyps'/exp OR 'chronic rhinosinusitis with nasal polyps' OR 'nose polyp'/exp OR 'nose polyp' OR 'nasal polyps'/exp OR 'nasal polyps' OR 'nasal polyp'/exp OR 'nasal polyp' OR 'polyp, nasal' OR 'polyp, nasal or polyps, nasal' OR 'polyps, nasal' OR 'nasal cavity polyp'/exp OR 'nasal cavity polyp' OR 'nasal polyposis'/exp OR 'nasal polyposis' OR 'nose polyposis'/exp OR 'nose polyposis' OR 'polyp of the nasal cavity'/exp OR 'polyp of the nasal cavity' OR 'polyp of the nose'/exp OR 'polyp of the nose' OR 'polyp, nose'/exp OR 'polyp, nose' OR 'polyposis nasi'/exp OR 'polyposis nasi' OR 'polyposis of the nose'/exp OR 'polyposis of the nose' OR 'sino-nasal polyp'/exp OR 'sino-nasal polyp'	17,321
#2	"Microbiota/exp" OR "Microbiota" OR "Microbiotas/exp" OR "Microbiotas" OR "Microbial Community/exp" OR "Microbial Community" OR "Community, Microbial/exp" OR "Community, Microbial" OR "Microbial Communities/exp" OR "Microbial Communities" OR "Microbial Community Composition/exp" OR "Microbial Community Composition" OR "Community Composition, Microbial/exp" OR "Community Composition, Microbial" OR "Composition, Microbial Community/exp" OR "Composition, Microbial Community" OR "Microbial Community Compositions/exp" OR "Microbial Community Compositions" OR "Microbial Community Structure/exp" OR "Microbial Community Structure" OR "Community Structure, Microbial/exp" OR "Community Structure, Microbial" OR "Microbial Community Structures/exp" OR "Microbial Community Structures" OR "Microbiome/exp" OR "Microbiome" OR "Microbiomes/exp" OR "Microbiomes" OR "Human Microbiome/exp" OR "Human Microbiome" OR "Human Microbiomes/exp" OR "Human Microbiomes" OR "Microbiome, Human/exp" OR "Microbiome, Human" OR "microflora/exp" OR "microflora" OR "microbial flora/exp" OR "microbial flora" OR "bacterial flora/exp" OR "bacterial flora" OR "bacterial microbiota/exp" OR "bacterial microbiota" OR "bacteriologic flora/exp" OR "bacteriologic flora" OR "bacterium flora/exp" OR "bacterium flora" OR "neonatal bacterial flora/exp" OR "neonatal bacterial flora" OR "bowel microbiota/exp" OR "bowel microbiota" OR "alimentary canal flora/exp" OR "alimentary canal flora" OR "alimentary tract flora/exp" OR "alimentary tract flora" OR "bowel flora/exp" OR "bowel flora" OR "digestive canal flora/exp" OR "digestive canal flora" OR "digestive tract flora/exp" OR "digestive tract flora" OR "enteric flora/exp" OR "enteric flora" OR "enteric microbiota/exp" OR "enteric microbiota" OR "flora intestine/exp" OR "flora intestine" OR "gastro intestinal flora/exp" OR "gastro intestinal flora" OR "gastrointestinal canal flora/exp" OR "gastrointestinal canal flora" OR "gastrointestinal flora/exp" OR "gastrointestinal flora" OR "gastrointestinal microbiome/exp" OR "gastrointestinal microbiome" OR "gastrointestinal microbiota/exp" OR "gastrointestinal microbiota" OR "gastrointestinal tract flora/exp" OR "gastrointestinal tract flora" OR "gastrointestine flora/exp" OR "gastrointestine flora" OR "gastrointestine tract flora/exp" OR "gastrointestine tract flora" OR "gut bacteria/exp" OR "gut bacteria" OR "gut microbiota/exp" OR "gut microbiota" OR	322,130

"intestinal bacteria/exp" OR "intestinal bacteria" OR "intestinal bacterial flora/exp" OR "intestinal bacterial flora" OR "intestinal bacterium/exp" OR "intestinal bacterium" OR "intestinal canal flora/exp" OR "intestinal canal flora" OR "intestinal flora/exp" OR "intestinal flora" OR "intestinal microbe/exp" OR "intestinal microbe" OR "intestinal microbes/exp" OR "intestinal microbes" OR "intestinal microbiota/exp" OR "intestinal microbiota" OR "intestinal microflora/exp" OR "intestinal microflora" OR "intestinal microorganism/exp" OR "intestinal microorganism" OR "intestinal tract flora/exp" OR "intestinal tract flora" OR "intestine bacteria/exp" OR "intestine bacteria" OR "intestine bacteria change/exp" OR "intestine bacteria change" OR "intestine bacterial flora/exp" OR "intestine bacterial flora" OR "intestine bacterium/exp" OR "intestine bacterium" OR "intestine microbial flora/exp" OR "intestine microbial flora" OR "intestine microflora/exp" OR "intestine microflora" OR "intestine flora/exp" OR "intestine flora" OR "host micro-organism interaction/exp" OR "host micro-organism interaction" OR "host microbial interaction/exp" OR "host microbial interaction" OR "host microbial interactions/exp" OR "host microbial interactions" OR "host microbiome interaction/exp" OR "host microbiome interaction" OR "host microbiota interaction/exp" OR "host microbiota interaction" OR "host microbiotal interaction/exp" OR "host microbiotal interaction" OR "host microorganism interaction/exp" OR "host microorganism interaction" OR "microbe host interaction/exp" OR "microbe host interaction" OR "microbial host interaction/exp" OR "microbial host interaction" OR "microbiome host interaction/exp" OR "microbiome host interaction" OR "microbiota host interaction/exp" OR "microbiota host interaction" OR "microorganism host interaction/exp" OR "microorganism host interaction" OR "host microbe interaction/exp" OR "host microbe interaction" OR "gingiva microflora/exp" OR "gingiva microflora" OR "mouth bacteria/exp" OR "mouth bacteria" OR "mouth bacterial flora/exp" OR "mouth bacterial flora" OR "mouth bacterium/exp" OR "mouth bacterium" OR "mouth microflora/exp" OR "mouth microflora" OR "oral bacteria/exp" OR "oral bacteria" OR "oral bacterial flora/exp" OR "oral bacterial flora" OR "oral bacterium/exp" OR "oral bacterium" OR "oral cavity flora/exp" OR "oral cavity flora" OR "oral flora/exp" OR "oral flora" OR "oral microbe/exp" OR "oral microbe" OR "oral microbes/exp" OR "oral microbes" OR "oral microbial flora/exp" OR "oral microbial flora" OR "oral microbiota/exp" OR "oral microbiota" OR "oral microflora/exp" OR "oral microflora" OR "oral microorganism/exp" OR "oral microorganism" OR "oral microorganisms/exp" OR "oral microorganisms" OR "throat flora/exp" OR "throat flora" OR "mouth flora/exp" OR "mouth flora" OR "lung flora/exp" OR "lung flora" OR "lung microbial community/exp" OR "lung microbial community" OR "lung microbiome/exp" OR "lung microbiome" OR "lung microflora/exp" OR "lung microflora" OR "lung microorganisms/exp" OR "lung microorganisms" OR "pulmonary flora/exp" OR "pulmonary flora" OR "pulmonary micro-organisms/exp" OR "pulmonary micro-organisms" OR "pulmonary microbial community/exp" OR "pulmonary microbial community" OR "pulmonary microbiome/exp" OR "pulmonary microbiome" OR "pulmonary microbiota/exp" OR "pulmonary microbiota" OR "pulmonary microflora/exp" OR "pulmonary microflora" OR "pulmonary microorganisms/exp" OR "pulmonary microorganisms" OR "lung microbiota/exp" OR "lung microbiota" OR "inter-microbial interaction/exp" OR "inter-microbial interaction" OR "microorganisms interaction/exp" OR "microorganisms interaction" OR "intermicrobial interaction/exp" OR "intermicrobial interaction" OR "microbe-microbe associations/exp" OR "microbe-microbe associations" OR "microbe-microbe interactions/exp" OR "microbe-microbe interactions" OR "microbe-microbe relationships/exp" OR "microbe-microbe relationships" OR "microbe-microbe sys-

	tem/exp" OR "microbe-microbe system" OR "microbial-microbial interactions/exp" OR "microbial-microbial interactions" OR "microbiota/microbiome interactions/exp" OR "microbiota/microbiome interactions" OR "microorganism interrelation/exp" OR "microorganism interrelation" OR "microorganism-microorganism interaction/exp" OR "microorganism-microorganism interaction" OR "microbe-microbe interaction/exp" OR "microbe-microbe interaction" OR "Dysbiosis/exp" OR "Dysbiosis" OR "Dysbioses/exp" OR "Dysbioses" OR "Disbiosis/exp" OR "Disbiosis" OR "Disbioses/exp" OR "Disbioses" OR "Dys-symbiosis/exp" OR "Dys-symbiosis" OR "Dys symbiosis/exp" OR "Dys symbiosis" OR "Dys-symbioses/exp" OR "Dys-symbioses" OR "Dysbacteriosis/exp" OR "Dysbacteriosis" OR "Dysbacterioses/exp" OR "Dysbacterioses" OR "Disbacteriosis/exp" OR "Disbacteriosis" OR "Disbacterioses/exp" OR "Disbacterioses" OR "Gastrointestinal Microbiomes/exp" OR "Gastrointestinal Microbiomes" OR "Microbiome, Gastrointestinal/exp" OR "Microbiome, Gastrointestinal" OR "Gut Microbiome/exp" OR "Gut microbiome" OR "Gut Microbiomes/exp" OR "Gut microbiomes" OR "Microbiome, Gut/exp" OR "Microbiome, Gut" OR "Gut Microflora/exp" OR "Gut microflora" OR "Microflora, Gut/exp" OR "Microflora, Gut" OR "Gut Microbiotas/exp" OR "Gut microbiotas" OR "Microbiota, Gut/exp" OR "Microbiota, Gut" OR "Flora, Gastrointestinal/exp" OR "Flora, Gastrointestinal" OR "Gut Flora/exp" OR "Gut flora" OR "Flora, Gut/exp" OR "Flora, Gut" OR "Gastrointestinal Microbiotas/exp" OR "Gastrointestinal Microbiotas" OR "Microbiota, Gastrointestinal/exp" OR "Microbiota, Gastrointestinal" OR "Gastrointestinal Microbial Community/exp" OR "Gastrointestinal Microbial Community" OR "Gastrointestinal Microbial Communities/exp" OR "Gastrointestinal Microbial Communities" OR "Microbial Community, Gastrointestinal/exp" OR "Microbial Community, Gastrointestinal" OR "Gastrointestinal Microflora/exp" OR "Gastrointestinal Microflora" OR "Microflora, Gastrointestinal/exp" OR "Microflora, Gastrointestinal" OR "Gastric Microbiome/exp" OR "Gastric Microbiome" OR "Gastric Microbiomes/exp" OR "Gastric Microbiomes" OR "Microbiome, Gastric/exp" OR "Microbiome, Gastric" OR "Intestinal Microbiome/exp" OR "Intestinal Microbiome" OR "Intestinal Microbiomes/exp" OR "Intestinal Microbiomes" OR "Microbiome, Intestinal/exp" OR "Microbiome, Intestinal" OR "Intestinal Microbiotas/exp" OR "Intestinal Microbiotas" OR "Microbiota, Intestinal/exp" OR "Microbiota, Intestinal" OR "Microflora, Intestinal/exp" OR "Microflora, Intestinal" OR "Flora, Intestinal/exp" OR "Flora, Intestinal" OR "Enteric Bacteria/exp" OR "Enteric Bacteria" OR "Bacteria, Enteric/exp" OR "Bacteria, Enteric" OR "Viromes/exp" OR "Viromes" OR "Virus Microbiome/exp" OR "Virus Microbiome" OR "Microbiome, Virus/exp" OR "Microbiome, Virus" OR "Microbiomes, Virus/exp" OR "Microbiomes, Virus" OR "Virus Microbiomes/exp" OR "Virus Microbiomes" OR "Viral Microbiome/exp" OR "Viral Microbiome" OR "Microbiome, Viral/exp" OR "Microbiome, Viral" OR "Microbiomes, Viral/exp" OR "Microbiomes, Viral" OR "Viral Microbiomes/exp" OR "Viral Microbiomes" OR "Phageome/exp" OR "Phageome" OR "Phageomes/exp" OR "Phageomes" OR "bacterial biome/exp" OR "bacbacterial biome" OR "bacteriobiome/exp" OR "bacteriobiome" OR "bacteriome/exp" OR "bacteriome" OR "bacterial microbiome/exp" OR "bacterial microbiome" OR "phagome/exp" OR "phagome" OR "viral biome/exp" OR "viral biome" OR "viral microbiome/exp" OR "viral microbiome" OR "virus microbiome/exp" OR "virus microbiome" OR "virome/exp" OR "virome"	
#3	#1 AND #2	227
